# Autophagy Inhibition–induced Cytosolic DNA Sensing Combined with Differentiation Therapy Induces Irreversible Myeloid Differentiation in Leukemia Cells

**DOI:** 10.1158/2767-9764.CRC-23-0507

**Published:** 2024-03-20

**Authors:** Tomohisa Baba, Utano Tomaru, Atsushi Hirao, Naofumi Mukaida, Yoshikazu Johmura

**Affiliations:** 1 Division of Cancer and Senescence Biology, Kanazawa University, Kanazawa, Japan.; 2 Department of Surgical Pathology, Hokkaido University Hospital, Sapporo, Japan.; 3 Division of Molecular Genetics, Cancer Research Institute, Kanazawa, Japan.; 4 Nano Life Science Institute, Kanazawa University, Kanazawa, Japan.; 5 Department of Forensic Medicine, Wakayama Medical University, Wakayama, Japan.

## Abstract

Accumulating evidence indicates that various oncogenic mutations interfere with normal myeloid differentiation of leukemogenic cells during the early process of acute myeloid leukemia (AML) development. Differentiation therapy is a therapeutic strategy capable of terminating leukemic expansion by reactivating the differentiation potential; however, the plasticity and instability of leukemia cells counteract the establishment of treatments aimed at irreversibly inducing and maintaining their differentiation states. On the basis of our previous observation that autophagy inhibitor treatment induces the accumulation of cytosolic DNA and activation of cytosolic DNA-sensor signaling selectively in leukemia cells, we herein examined the synergistic effect of cytosolic DNA-sensor signaling activation with conventional differentiation therapy on AML. The combined treatment succeeded in inducing irreversible differentiation in AML cell lines. Mechanistically, cytosolic DNA was sensed by absent in melanoma 2 (AIM2), a cytosolic DNA sensor. Activation of the AIM2 inflammasome resulted in the accumulation of p21 through the inhibition of its proteasomal degradation, thereby facilitating the myeloid differentiation. Importantly, the combined therapy dramatically reduced the total leukemia cell counts and proportion of blast cells in the spleens of AML mice. Collectively, these findings indicate that the autophagy inhibition-cytosolic DNA-sensor signaling axis can potentiate AML differentiation therapy.

**Significance:**

Clinical effects on AML therapy are closely associated with reactivating the normal myeloid differentiation potential in leukemia cells. This study shows that autophagosome formation inhibitors activate the cytosolic DNA-sensor signaling, thereby augmenting conventional differentiation therapy to induce irreversible differentiation and cell growth arrest in several types of AML cell lines.

## Introduction

Acute myeloid leukemia (AML) is a heterogenous hematopoietic malignancy caused by various oncogenic mutations. However, accumulating evidence suggests that many genetic and epigenetic alterations commonly block normal myeloid differentiation of immature states of leukemia cells (1). In particular, *PML-RARA gene*, resulting from the t(15;17) translocation, drives abnormal expansion of leukemia cells by inhibiting normal myeloid differentiation through retinoic acid receptor (RAR) signaling in acute promyelocytic leukemia (APL; refs. 2, 3). In contrast, all-trans retinoic acid (ATRA), which activates RAR signaling and degrades PML-RARA (4), can effectively drive the myeloid differentiation and inhibit the proliferation of leukemia cells (5–8). Thus, the therapeutic treatment of pharmacologic agents, which succeeded in ameliorating the prognosis of APL by promoting the differentiation of immature leukemia cells, have been investigated and is termed as “differentiation therapy” (1, 6). At secondary relapse, however, some cases presented with resistance to ATRA or mature leukemia cells whose leukemogenic property had been regained through dedifferentiation, especially in patients treated with low-dose consolidation (9, 10). Although ATRA can promote differentiation in some types of non-APL AML cells by activating the normal RAR *in vitro*, it is generally ineffective in patients with non-APL AML (11–14). Meanwhile, inhibition of lysine-specific demethylase 1 or its combined inhibition with histone acetyltransferase, GCN5, can potentiate ATRA-driven therapeutic response in non-APL AML (15, 16). Moreover, it was reported that insulin-like growth factor binding protein 7 downregulated the transcription factor GFI1, thereby augmenting ATRA-mediated reduction of AML stem/progenitor cells (17). Furthermore, leukemia cells progress toward matured states in patients treated with molecular targeted therapies against several driver mutations including mutant isocitrate dehydrogenase (IDH)1, IDH2, and FLT3, assuming that the concept of differentiation therapy is also applicable to non-APL (1). Therefore, effective interventions enabling irreversible differentiation in various types of myeloid leukemia potentially improve their clinical prognosis.

Cytosolic double-stranded (ds)DNA is released from nuclei with a compromised integrity and can be recognized by various sensors as a damage-associated molecular pattern. Cytosolic DNA-sensors are functionally divided into those concerning type Ⅰ IFN production and those concerning inflammasome activation (18). In the former group, a well-studied DNA-sensor is cyclic GMP-AMP (cGAMP) synthase (cGAS), which produces cGAMP after sensing dsDNA in the cytoplasm (19, 20). Then, cGAMP is recognized by a central adaptor molecule, stimulator of IFN genes (STING), leading to type I IFN production (21). On the other hand, a representative inflammasome-activating DNA-sensor, AIM2, can induce IL1β maturation and pyroptosis, a type of immunogenic cell death, via enzymatic digestion by caspase-1 (22–25). Cytosolic DNA accumulation is preferentially observed in various types of malignant cells and can be ascribed to DNA release from multiple origins (26). However, the biological effects of cytosolic DNA in cancer remain elusive.

We previously observed that myeloid leukemia cells constitutively leak dsDNA into the cytoplasm, probably due to uncontrolled cell cycle progression and subsequent genome instability (27). A detailed analysis of cytosolic DNA dynamics revealed that cytosolic DNA can be degraded in leukemia cells through autophagy. Furthermore, inhibition of autophagosome formation, the early autophagy event, but not autolysosome formation, the late event, results in cytosolic dsDNA accumulation, activating cytosolic DNA-sensor signaling pathways. Thus, autophagy inhibition–induced accumulation of cytosolic DNA can be an effective treatment approach against AML. Here, we examined the potential synergistic effect of cytosolic DNA-sensor signaling activation and conventional differentiation therapy on various types of AML cell lines. Of interest, short-term treatment with the combined therapy with autophagy inhibitors and ATRA sufficiently induced irreversible myeloid differentiation in AML cells by activating the cytosolic DNA-sensor, AIM2.

## Materials and Methods

### Mice

Specific pathogen-free 6 to 8 weeks old male BALB/c mice were purchased from Charles River Japan. All mice were kept under specific pathogen-free conditions, housed (five per cage) in a temperature (21°C–25°C), and maintained on a 12-hour light/dark cycle (08:00 to 20:00 lights on). Mice were provided standard food and water *ad libitum*. Animal studies were approved by the Committee of Laboratory Animals of Kanazawa University (Kanazawa, Japan; AP-214222). All experiments were performed in accordance with relevant guidelines and regulations and the authors complied with the ARRIVE (Animal Research: Reporting of In Vivo Experiments) guidelines.

### Abs

The following rat anti-mouse mAbs; anti-CD11b (M1/70, TONBO Biosciences) and anti-CD19 (1D3, TONBO Biosciences), and mouse or rabbit anti-human mAbs; anti-CD38 (HB7, TONBO Biosciences), anti-p21 (12D1, Cell Signaling Technology), anti-AIM2 (3B10, BioLegend), anti-SKP2 [EPR3305(2), Abcam], and anti-USP10 (D7A5, Cell Signaling Technology) were used. Isotype-matched control IgGs for individual rat and mouse mAb were purchased from BD Biosciences. PE-conjugated donkey anti-rabbit IgG (Thermo Fisher Scientific) was used as the secondary Ab for the flow cytometry analysis.

### Culture of Cell Lines and Cell Repopulation Assay

The human AML cell lines, MOLM-13, MOLM-14, and KG-1 cell lines were obtained from JCRB Cell Bank. The HL-60 and THP-1 cell lines were purchased from ATCC. The human CML cell line, K562, was obtained from RIKEN BRC Cell Bank. The authentication of purchased cell lines was performed by the respective cell bank, and further authentication was not conducted. The experiments utilized frozen stocks of cell lines, which were prepared within a few passages after acquisition. A *Mycoplasma* test was not conducted upon receipt from the cell bank. All cell lines were cultured in RPMI1640 medium supplemented with 10% FBS. In the repopulation assay, cells were washed twice with fresh culture medium at the indicated time periods of drug treatment. Then, viable cells were counted using trypan blue staining method and replated in 96-well plates (10^4^ viable cells/well). Cell proliferation was determined using a cell counting kit-8 (DOJINDO) at the indicated timepoints until confluency was reached. In certain experiments, cells cultured in low-adherent culture dishes (EZ-BindShut, IWAKI) to inhibit the adhesion of differentiated leukemia cells were subjected to each analysis.

### Establishment of Knockdown Cell Lines

STING and AIM2 knockdown (KD) cells were previously established from HL-60 or THP-1 cell lines, and their KD efficiencies were determined (27): shSTING-1 HL-60, 83.1%; shSTING-2 HL-60, 83.3%; shAIM2-1 HL-60, 93.1%; shAIM2-2 HL-60, 91.8%; shAIM2-1 THP-1, 73.1%; shAIM2-2 THP-1, 60.1%. AIM2 KD MOLM-14 cell lines were newly established by infection with MISSION lentivirus carrying pLKO.1 puro with short hairpin RNA (shRNA) constructs (AIM2-1: TRCN0000107503, AIM2-2: TRCN0000107504), which were purchased from Sigma-Aldrich. To establish p21 KD cells, HL-60 cells were infected with MISSION lentivirus carrying pLKO.1 puro with shRNA constructs (p21-1: TRCN0000040123, p21-2: TRCN0000287021). As a control, each cell line was infected with MISSION lentivirus carrying pLKO.1 puro with non-mammalian shRNA control constructs. Stably transfected cells were selected in culture medium supplemented with 1 µg/mL puromycin for more than 2 weeks.

### Retroviral Preparation

The MSCV-MLL-AF9-ires-GFP vector was a gift from Akihiko Yokoyama, National Cancer Center, Tsuruoka Metabolomics Laboratory, Japan. Retroviral packaging cells (Phoenix 293T) were transiently transfected with the MSCV-MLL-AF9-ires-GFP plasmid using jetPRIME transfection reagent (Polyplus-transfection) to produce the retrovirus carrying MSCV-MLL-AF9-ires-GFP in the culture supernatant, which was subsequently used to infect lineage^−^c-kit^+^sca-1^+^ (LKS^+^) cells isolated from mouse bone marrow (BM).

### Generation of Mouse AML Model

LKS^+^ cells purified from the BM of BALB/c mice were cultured in serum-free S-Clone SF-03 medium (Sanko Junyaku) supplemented with 1% BSA, 100 ng/mL stem cell factor (PeproTech), 100 ng/mL thrombopoietin, 25 ng/mL *fms*-like tyrosine kinase-3 ligand (PeproTech), 10 ng/mL IL6 (PeproTech), and 10 ng/mL IL3 (PeproTech) for 24 hours. Cultured LKS^+^ cells were infected with the retrovirus carrying MSCV-MLL-AF9-ires-GFP using a ViroMag R/L kit (OZ Bioscience) to obtain leukemia-initiating cells. The resultant cells (50–100 GFP^+^ cells included in 30,000 KLS^+^ cells) were intravenously transplanted into 5 Gy X-irradiated recipient BALB/c mice along with 3 × 10^5^ normal BM cells in a 200 µL volume. Total BM cells were harvested from primary AML mice and stored in liquid nitrogen. A frozen stock of primary AML cells was cultured in semisolid methylcellulose-based medium (Methocult GF M3534, STEMCELL technologies). To prepare the mouse AML model, 2 × 10^5^ colony-forming cells were intravenously transplanted into sublethally irradiated secondary recipient mice along with 1 × 10^6^ normal BM cells.

### *In Vivo* Drug Administration

ATRA (Sigma-Aldrich) and MRT-68921 (MRT; Sigma-Aldrich) were dissolved in DMSO and distilled water to make a 2.5 and 20 mg/mL stock solution, respectively. Mice bearing AML were intraperitoneally injected with ATRA [5 mg/kg, diluted in PBS containing 5% polyethylene glycol 400 (Sigma-Aldrich) and 5% Tween 80 (Sigma-Aldrich)] or MRT (20 mg/kg, diluted in PBS).

### Nitro Blue Tetrazolium Reduction Assay

One nitro blue tetrazolium (NBT) tablet (Sigma-Aldrich) was dissolved in 10 mL culture medium and used as the working solution. Cells were incubated with 10 µg/mL phorbol 12-myristate 13-acetate in the NBT working solution for 20 minutes at 37°C. Then, their cytospin-prepared slides were fixed with methanol and counterstained with 0.15% safranin O for 30 seconds.

### Extraction and Measurement of Cytosolic dsDNA

The cytoplasmic fraction of leukemia cells was extracted using a cell fractionation kit (Abcam). The concentration of dsDNA was specifically measured with Qubit 4 (Thermo Fisher Scientific) using a Qubit 1X dsDNA HS assay kit (Thermo Fisher Scientific).

### RNA Extraction, cDNA Synthesis, and qRT-PCR

Total RNAs were extracted from cells using an RNeasy Mini Kit (QIAGEN) and then reverse-transcribed using the SuperScript IV VILO (Thermo Fisher Scientific). qRT-PCR was performed on StepOne Real-time PCR system (Thermo Fisher Scientific) using the Luna Universal qPCR Master Mix (New England BioLabs). The following specific primer sets were used: human *CXCL3* gene (sense: 5′-ACC GAA GTC ATA GCC ACA CTC-3′; antisense: 5′-AGT TGG TGC TCC CCT TGT T-3′), human *GAPDH* gene (sense: 5′-GCC AAA AGG GTC ATC TC-3′; antisense: 5′-TGA GTC CTT CCA CGA TAC CA-3′), human *NFκB2* gene (sense: 5′-CTC GAA TGG ACA AGA CAG CA-3′; antisense: 5′-TTA CAG GCC GCT CAA TCT TC-3′), human *P21* gene (sense: 5′-AGG GGA CAG CAG AGG AAG AC-3′; antisense: 5′-GGC GTT TGG AGT GGT AGA AA-3′), human *RRM2* gene (sense: 5′-CTG GCT CAA GAA ACG AGG AC-3′; antisense: 5′-GTT TGA ACA TCA GGC AAG CA-3′), human *TNFα* gene (sense: 5′-GGC GTG GAG CTG AGA GAT AA-3′; antisense: 5′-GAT GGC AGA GAG GAG GTT GA-3′), human *TYMS* gene (sense: 5′-TCC CGA GAC TTT TTG GAC AG-3′; antisense: 5′-TCA GGG TTG GTT TTG ATG GT-3′). The relative expression of each gene was analyzed by the ΔΔCt method using the Ct value of the *GAPDH* gene.

### Flow Cytometry

Intracellular p21, AIM2, SKP2, and USP10 were stained with each protein-specific mAb using a Foxp3/Transcription Factor Buffer Set (eBioscience). For apoptosis and cell cycle analyses, cells were stained using Annexin V-FITC Apop kit (Thermo Fisher Scientific) and Vybrant DyeCycle Green (Thermo Fisher Scientific), respectively. The expression of each molecule was determined using a FACSCantoII (BD Biosciences) and analyzed with FlowJo software (Tree Star).

### Immunoprecipitation and Western Blot Analysis for the Detection of Ubiquitylated p21

Cells were lysed in CelLytic M (Sigma) containing 1% Proteoguard (Clontech) for 15 minutes at 4°C. The supernatants were harvested after centrifugation at 15,000 × *g* for 15 minutes and subjected to immunoprecipitation (IP) to isolate the p21 protein using a rabbit anti-human p21 mAb (E2R7A, Cell Signaling Technology) and a Dynabeads Protein G IP kit (Thermo Fisher Scientific) according to the manufacturer's instructions. The resultant IP samples were subjected to SDS-PAGE and blotted onto nitrocellulose membranes. The blots were probed with rabbit anti-p21 mAb (E2R7A) or mouse anti-ubiquitin mAb (UBCJ2, Enzo Life Sciences) and reacted with horseradish peroxidase–conjugated anti-rabbit or anti-mouse IgG (Jackson ImmunoResearch). The immune complexes were visualized using ImmunoStar LD (FUJIFILM Wako Chemicals) and detected with Imager 680 (Cytiva). Signal intensities were analyzed by ImageJ software. As a negative control, we confirmed that the GAPDH protein was hardly detected in any of the IP samples.

### Microarray Analysis

Total RNA was extracted and its quality was checked using an Agilent Bioanalyzer (Agilent Technologies). The cDNA was synthesized using a GeneChip Whole Transcript Amplification kit (Thermo Fisher Scientific). The sense cDNA was then fragmented and biotin-labeled with terminal deoxynucleotidyl transferase using a GeneChip WT Terminal Labeling kit (Thermo Fisher Scientific). Labeled DNA (5.5 µg) was hybridized to the Affymetrix GeneChip Array (Clariom S Assay human, Thermo Fisher Scientific) at 45°C for 16 hours. Slides were washed, stained on a GeneChip Fluidics Station 450 (Thermo Fisher Scientific), and scanned with a GCS3000 Scanner (Thermo Fisher Scientific). The probe cell intensity data computation and CEL file generation were performed using Affymetrix GeneChip Command Console software (Thermo Fisher Scientific). The obtained data were normalized and filtered using Affymetrix Power Tools (Thermo Fisher Scientific), and then subjected to gene set enrichment analysis (GSEA). Differentially expressed genes (fold change ≥ 3) were subjected to Gene Ontology functional analysis (data source category: Biological process) using gProfiler. GSEA was executed through GenePattern server. As the metric for ranking genes, the log_2__ratio_of_means was exploited, and the gene set size was set to 15–500. Hallmark gene sets were used in this study.

### Statistical Analysis

We did not use specific sample size calculation methods. Any technically validated data were not excluded. Data were analyzed statistically using GraphPad Prism software (Ver. 6) using the methods indicated in the legend of each figure. Two-sided Student *t* test and one-way ANOVA followed by Dunnett or Tukey-Kramer *post hoc* test was used to compare the data among two and more than two groups, respectively. The log-rank test was used to evaluate the survival curve data. Statistical significance was set at *P* < 0.05.

### Data Availability

Raw and processed microarray data are available at Gene Expression Omnibus (GEO) under accession number GSE193056 (GEO reviewer link: https://www.ncbi.nlm.nih.gov/geo/query/acc.cgi?acc=GSE193056).

## Results

### Combined Treatment with Autophagosome Formation Inhibitors and ATRA Induces Irreversible Differentiation in Myeloid Leukemia Cells

**FIGURE 1:**
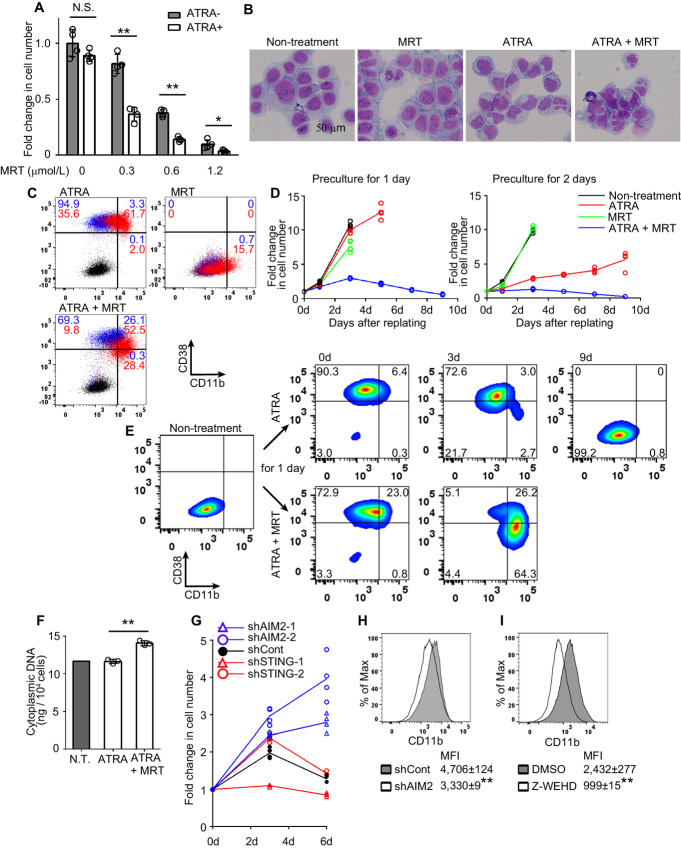
Combined treatment with MRT and ATRA induces the irreversible differentiation of human leukemia cells by activating the AIM2 inflammasome. **A,** HL-60 cells were treated with the indicated concentrations of MRT in the presence or absence of 1 µmol/L ATRA for 48 hours. Fold change in cell number was calculated by dividing the values of the treated samples by the mean value of the untreated control. Data represent the mean ± SD from four independent experiments. **B,** Giemsa staining of HL-60 cells 48 hours after combined or single treatment with 1 µmol/L ATRA and 1 µmol/L MRT. Representative results from three independent experiments are shown here. **C,** Expression of CD38 and CD11b on untreated (black dots) and treated [with ATRA, MRT, or ATRA+MRT for 24 hours (blue dots) or 48 hours (red dots)] HL-60 cells. Percentage of each quadrant is shown (24 hours, blue; 48 hours, red). Representative results from three independent experiments are shown here. **D,** Cell growth in drug-free medium after prior incubation with ATRA, MRT, or ATRA+MRT for 24 and 48 hours (*n* = 4). Fold change in cell number was calculated by dividing the values at each timepoint with the values at 0d. **E,** Alteration of CD38 and CD11b expression in HL-60 cells. Surface phenotypes were determined at the indicated timepoints after replating in drug-free medium following incubation with ATRA or ATRA+MRT for 24 hours. Representative results from three independent experiments are shown here. **F,** Cytoplasmic dsDNA in HL-60 cells 18 hours after combined or single treatment with 1 µmol/L ATRA and 1 µmol/L MRT. Data represent the mean ± SD from three independent experiments. Single data of non-treated cells are shown as a basement level of cytoplasmic dsDNA. **G,** Cell proliferation of shControl-, shSTING-, and shAIM2-transduced HL-60 cells in drug-free medium after 24 hours pre-exposure to ATRA+MRT (*n* = 4). Fold change in cell number was calculated by dividing the values at each timepoint with the values at 0d. **H,** Expression of CD11b on shControl- and shAIM2-transduced HL-60 cells after ATRA+MRT treatment for 24 hours. Representative results and mean ± SD of mean fluorescent intensities (MFI) from three independent experiments are shown here. **I,** Expression of CD11b on HL-60 cells after ATRA+MRT treatment in the presence of 100 µmol/L Z-WEHD-FMK (BioVision) or DMSO (control) for 24 hours. Representative results and mean ± SD of MFI from three independent experiments are shown here. **, *P* < 0.01; *, *P* < 0.05 using two-sided Student *t* test. d, day.

We previously reported that Atg5-specific siRNA treatment and autophagosome formation inhibitors, MRT and SBI-0206965 (SBI), but not an autolysosome inhibitor, hydroxychloroquine, induced accumulation of cytosolic DNA fragments preferentially in leukemia cells (27). This prompted us to examine the effects of MRT on ATRA-mediated cell proliferation inhibition in HL-60 cells (28), which are myeloid leukemia cells without PML-RARA mutation. Indeed, MRT synergistically suppressed the proliferation of HL-60 cells in combination with ATRA (Fig. 1A). Moreover, combined treatment with ATRA and MRT (ATRA+MRT), but not monotherapy with either ATRA or MRT, induced nuclear indentation or segmentation in HL-60 cells, which is characteristic of myeloid differentiation (Fig. 1B). ATRA+MRT rapidly induced the expression of an early myeloid cell differentiation marker, CD38, to the same extent as that induced by ATRA alone; however, only ATRA+MRT induced decreased CD38 expression in CD11b^high^ cells (Fig. 1C), a late differentiation phenotype (29). Furthermore, 24- and 48-hour pre-exposure to ATRA+MRT irreversibly prevented cell proliferation even after the drug was washed off (subsequently referred to as irreversible cell growth arrest), whereas pre-exposure to either ATRA or MRT failed to do so (Fig. 1D). Given the observation that ATRA+MRT hardly induced apoptotic cell death (Supplementary Fig. S1), we assumed that this treatment mainly induced myeloid cell differentiation, thereby irreversibly arresting cell growth. Consistently, HL-60 cells treated with ATRA+MRT for 24 hours continuously progressed toward a late differentiation phenotype and eventually died under the same condition, whereas cells pretreated with ATRA alone only showed transiently enhanced CD38 expression and finally exhibited a CD38^low^CD11b^medium^ phenotype, which was similar to that of untreated cells (Fig. 1E). In addition, ATRA+SBI induced myeloid differentiation to the same extent as ATRA+MRT, as evidenced by increased NBT staining, and eventually caused irreversible cell growth arrest (Supplementary Fig. S2). Similar cell growth arrest was observed when other myeloid leukemia cell lines (THP-1, K562, and KG-1) were treated with ATRA+MRT (Supplementary Fig. S3). On the contrary, the monotherapy with autophagy inhibitors induced neither myeloid cell differentiation nor irreversible cell growth arrest in AML cell lines (Supplementary Fig. S3; Fig. 1B–D). Thus, these observations suggest that autophagosome formation inhibitors can induce irreversible myeloid differentiation only when combined with ATRA, and therefore prompted us to focus on the combined effects of MRT on the conventional differentiation therapy in our subsequent experiments.

### The AIM2 Inflammasome Drives Irreversible Differentiation in Leukemia Cells

ATRA+MRT, but not ATRA, markedly increased the content of cytosolic dsDNA in HL-60 cells (Fig. 1F), confirming our previous observation of cytosolic dsDNA accumulation by autophagosome formation inhibitors (27). Because the AIM2 and STING pathways are major cytosolic DNA-sensor signaling pathways (18), each of the molecules was silenced with the use of shRNAs in HL-60 cells to address their contribution. AIM2-specific shRNA transduction, but not STING-specific, rescued the ATRA+MRT-mediated irreversible cell proliferation blockage (Fig. 1G) and decreased CD11b expression (Fig. 1H). Among two AIM2 KD cell lines, shAIM2-2 exhibiting more efficient recovery of cell growth was mainly subjected to the subsequent experiments as shAIM2. Cytosolic DNA sensing induces AIM2 inflammasome to activate an inflammatory caspase, caspase-1, and thereby exerting biological functions such as pyroptotic cell death (25). Hence, we next explored the roles of caspase-1 in this process. The increased expression of CD11b induced by ATRA+MRT was reduced by treatment with a caspase-1 inhibitor, Z-WEHD (Fig. 1I). Thus, the AIM2-caspase-1 pathway drives ATRA+MRT-induced irreversible differentiation in myeloid leukemia cells.

### The Involvement of p21 Proteasomal Degradation Inhibition in AIM2-mediated Myeloid Differentiation of Leukemia Cells

**FIGURE 2:**
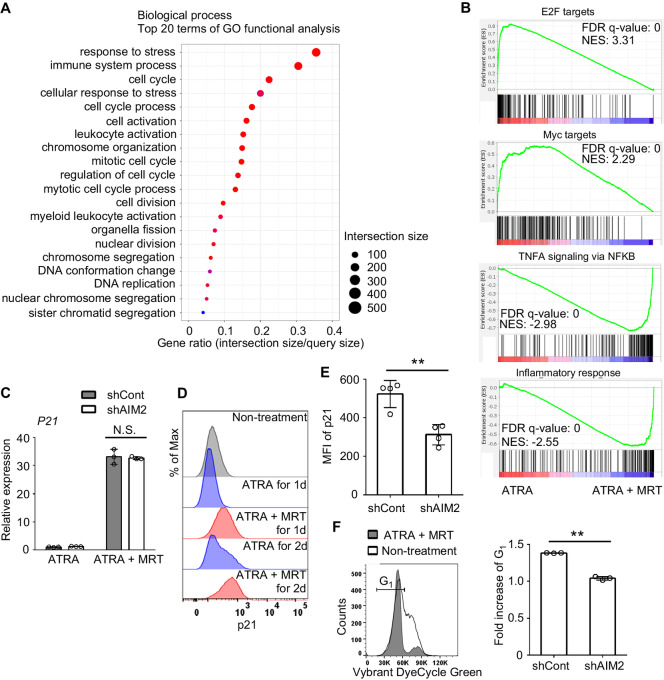
The AIM2 inflammasome is responsible for the accumulation of the p21 protein and regulation of cell cycle progression. **A,** GO functional analysis of differentially expressed genes between HL-60 cells treated with 1 µmol/L ATRA and 1 µmol/L MRT and cells treated with ATRA alone for 48 hours. The result from a single experiment is shown. **B,** Here, we show that the representative gene sets, which were identified with GSEA using hallmark gene sets among differentially regulated gene sets, were related to cell cycle and leukocyte activation. FDR and normalized enrichment score (NES) values were calculated from a single-sample experiment. **C,** Relative mRNA expression of p21 in shControl- and shAIM2-transduced HL-60 cells 24 hours after ATRA+MRT or ATRA treatment. Data represent the mean ± SD from three independent experiments. **D,** Expression of p21 protein 24 and 48 hours after ATRA+MRT or ATRA treatment. Non-treated cells were used as a control. Representative results from three independent experiments are shown here. **E,** Expression of the p21 protein in shControl- and shAIM2-transduced HL-60 cells 24 hours after ATRA+MRT treatment. Data represent the mean ± SD from four independent experiments. **F,** Change of cell cycle status 24 hours after ATRA+MRT treatment. The left panel demonstrates representative data of cell cycle status in shControl-transduced HL-60 cells either untreated or treated with ATRA+MRT. shControl- and shAIM2-transduced HL-60 cells were either untreated or treated with ATRA+MRT. Fold increases in G_1_-phase were calculated on shControl- or shAIM2-transduced HL-60 cells by dividing the percentage of cells in G_1_-phase of ATRA+MRT-treated cells with that of untreated cells, and are shown in the right. Data represent the mean ± SD from three independent experiments. **, *P* < 0.01; N.S., no significant difference using two-sided Student *t* test. GO, Gene Ontology.

**FIGURE 3:**
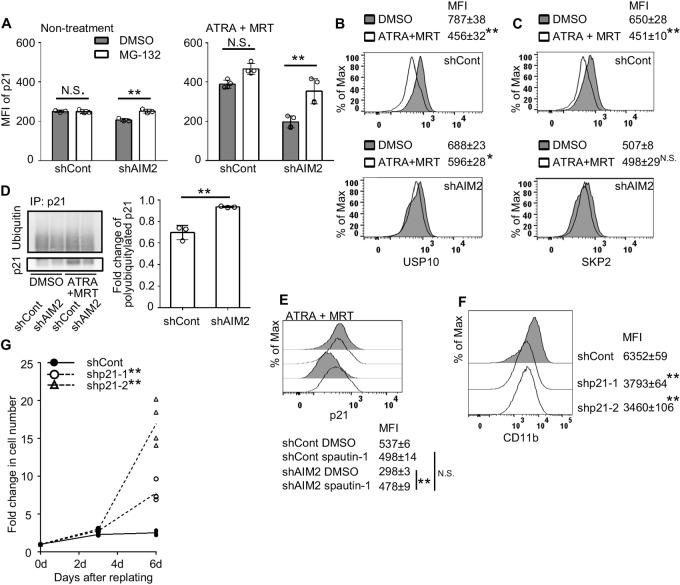
Involvement of the AIM2 inflammasome in leukemic cell differentiation through the inhibition of p21 proteasomal degradation and subsequent p21 accumulation. **A,** Expression of p21 in shControl- and shAIM2-transduced HL-60 cells 24 hours after incubation with or without 1 µmol/L ATRA and 1 µmol/L MRT in the presence of 100 nmol/L MG-132 (Fujifilm Wako Chemicals) or DMSO (control). Data represent the mean ± SD from three independent experiments. Expression of USP10 (**B**) and SKP2 (**C**) in shControl- and shAIM2-transduced HL-60 cells treated with ATRA+MRT or DMSO (control) for 24 hours. Representative results and mean ± SD of MFI from three independent experiments are shown here. **D,** The levels of polyubiquitylated p21 protein, immunoprecipitated with anti-p21 mAb, were determined in HL-60 cells transduced with shControl and shAIM2, and treated with ATRA+MRT or DMSO (control) for 24 hours. Polyubiquitylated p21 was detected with the use of immunoblotting and a representative result is shown in the left. Intensities in polyubiquitylated p21 were determined, and ubiquitin ratios were calculated as the signal intensities of polyubiquitin divided by that of p21. Fold change in the levels of polyubiquitylated p21 was calculated by dividing the values in treated samples with the values in the DMSO control, and is shown in the right. Data represent the mean ± SD from three independent experiments. **E,** Expression of p21 in shControl- and shAIM2-transduced HL-60 cells after ATRA+MRT treatment in the presence of 2.5 µmol/L spautin-1 (MedChemExpress) or DMSO (control) for 24 hours. Representative results and mean ± SD of MFI from three independent experiments are shown here. **F,** Expression of CD11b on shControl- and shp21-transduced HL-60 cells after ATRA+MRT treatment for 24 hours. Representative results and mean ± SD of MFI from three independent experiments are shown here. **G,** Cell proliferation of shControl- and shp21-transduced HL-60 cells in drug-free medium after ATRA+MRT treatment for 24 hours (*n* = 4). Fold change in cell number was calculated by dividing the values at each timepoint with the values at 0d. **, *P* < 0.01; *, *P* < 0.05; N.S., no significant difference using Tukey-Kramer test (A and E), two-sided Student *t* test (B–D), or Dunnett test (F and G). d, day.

Consistent with the functional observations, transcriptomic analysis revealed augmented expression of leukocyte activation-related genes in differentiated HL-60 cells treated with ATRA+MRT, whereas the expression of cell cycle–related genes was decreased (Fig. 2A and B). qRT-PCR confirmed that the changes in the expression of representative genes among each gene set were consistent with the results of the transcriptomic analysis (Supplementary Fig. S4). In addition, AIM2 KD significantly modulated the changes in mRNA levels of these differentially expressed genes (Supplementary Fig. S5), except for those of the *p21* gene (Fig. 2C), the gene presumed to be essential for ATRA-mediated myeloid differentiation in APL cells (30). In fact, combined treatment induced a greater increase in p21 protein levels than ATRA monotherapy (Fig. 2D), and the increment was significantly inhibited in AIM2 KD (Fig. 2E). This inhibition of p21 protein but not its mRNA expression was similarly observed in another AIM2 KD cell line (Supplementary Fig. S6). Likewise, AIM2 shRNA treatment reduced the ATRA+MRT-mediated upregulation of CD11b and p21 protein expression in THP-1 cells, another human leukemia cell line (Supplementary Fig. S7). Furthermore, ATRA+MRT treatment induced G_1_ arrest in HL-60 cells, and this G_1_ arrest was released by AIM2 KD (Fig. 2F). These findings suggest that the AIM2 inflammasome can control p21 protein levels at a posttranslational level, thereby regulating cell cycle progression. Indeed, the AIM2 KD-induced decrease in p21 protein levels was abrogated by a proteasome inhibitor, MG-132 (Fig. 3A). Thus, the AIM2 inflammasome can regulate p21 proteasomal degradation, which is presumed to be tightly controlled by SCF-SKP2 E3 ligase complex-mediated ubiquitylation (31). SKP2 is itself regulated by ubiquitylation and can be stabilized by a deubiquitinase, USP10 (32), a potential substrate of caspase-1 (33). ATRA+MRT treatment reduced both USP10 and SKP2 protein levels as well as the ratio of polyubiquitylated p21, while these decreases were abrogated by AIM2 KD (Fig. 3B–D). Furthermore, a USP10 inhibitor, spautin-1, markedly attenuated the AIM2 KD-mediated decrease in p21 protein levels (Fig. 3E). Similarly, p21 KD (Supplementary Fig. S8) attenuated the ATRA+MRT-induced enhancement of CD11b expression (Fig. 3F) and irreversible cell growth arrest (Fig. 3G). Together, the results demonstrated that ATRA+MRT treatment can activate the AIM2 inflammasome-caspase-1 pathway to inhibit p21 proteasomal degradation, promoting myeloid differentiation and cell growth arrest in leukemia cells.

### Autophagy Inhibitors Together with an FLT3 Inhibitor Synergistically Induces Irreversible Differentiation in Leukemia Cells with the FLT3 Mutation

**FIGURE 4:**
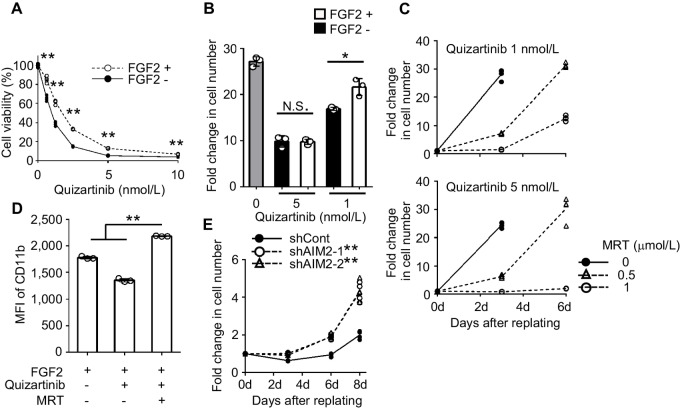
Synergistic effect of MRT and FLT3 inhibitor on leukemia cells carrying FLT3-ITD. **A,** MOLM-14 cells were treated with quizartinib (ChemScene) in the presence or absence of 10 ng/mL FGF2 (ProteinTech) for 48 hours (*n* = 4). Cell viability was calculated by dividing the values in treated cells with the mean values of the quizartinib-free control. **B,** Cell proliferation of MOLM-14 cells in drug-free medium after treatment with quizartinib in the presence or absence of FGF2 for 24 hours. Fold change in cell number was calculated by dividing the values at 3d with the values at 0d. Data represent the mean ± SD from three independent experiments. **C,** Cell proliferation of MOLM-14 cells in drug-free medium after combined treatment with quizartinib and MRT in the presence of FGF2 for 24 hours (*n* = 4). Fold change in cell number was calculated by dividing the values at each timepoint with the values at 0d. **D,** Expression of CD11b on MOLM-14 cells after treatment with 5 nmol/L quizartinib and 1 µmol/L MRT in the presence of FGF2 for 24 hours. MFI of cells stained with APC-conjugated anti-CD11b mAb are shown. Data represent the mean ± SD from three independent experiments. **E,** Cell proliferation of shControl- and shAIM2-transduced MOLM-14 cells in drug-free medium after combined treatment with quizartinib and MRT in the presence of FGF2 for 24 hours (*n* = 4). Fold change in cell number was calculated by dividing the values at each timepoint with the values at 0d. **, *P* < 0.01; *, *P* < 0.05; N.S., no significant difference using two-sided Student *t* test (A and B), Tukey-Kramer test (D), or Dunnett test (E). d, day.

In a clinical study, FLT3 inhibitors effectively reduced the numbers of leukemia blast cells in peripheral blood (PB), but not those in the BM, where the cells were relatively resistant to these drugs (34). This drug resistance can be partially ascribed to stromal cell-derived FGF2 (35). Consistently, FGF2 blunted the cytotoxicity and retardation of cell proliferation induced by the FLT3 inhibitor, quizartinib, after drug removal in MOLM-14, a human AML cell line carrying an FLT3 internal tandem duplication mutation (FLT3-ITD; Fig. 4A and B). However, combined incubation with quizartinib and MRT blocked cell repopulation in a dose-dependent manner even in the presence of FGF2 (Fig. 4C). The same treatment further induced upregulation of CD11b expression (Fig. 4D) and emergence of NBT^+^ cells with segmented nuclei (Supplementary Fig. S9). Moreover, AIM2 KD inhibited the increase in p21 protein levels, appearance of cells with segmented nuclei, and cell growth arrest in MOLM-14 cells treated with quizartinib and MRT (Supplementary Fig. S10; Fig. 4E). Thus, the autophagosome formation inhibition-mediated activation of AIM2 inflammasome can also synergize with a molecular-targeted drug, FLT3 inhibitor, to induce irreversible differentiation in leukemia cells with FLT3-ITD even in the presence of FGF2. In addition, quizartinib+MRT induced myeloid differentiation and cell growth arrest more efficiently than quizartinib monotherapy in the absence of FGF2 (Supplementary Fig. S11).

### Therapeutic Effects of Short-term Treatment with ATRA and MRT in the AML Mouse Model

**FIGURE 5:**
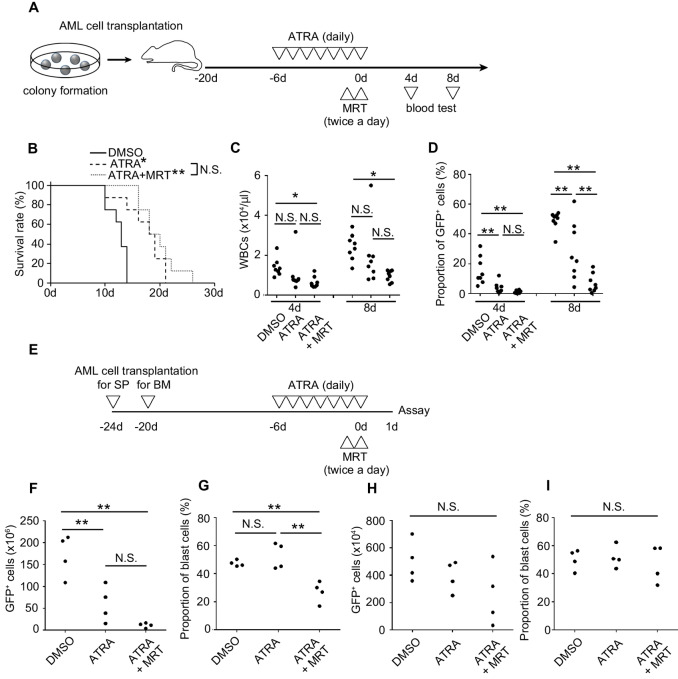
Combined treatment with ATRA and MRT retards the progression of AML *in vivo*. **A,** Schematic representation of the experimental procedure of combined treatment with ATRA and MRT in mice bearing AML. As a control, mice were treated with a corresponding vehicle. **B,** Survival rates of mice bearing AML (8 mice per group). Total WBC count (**C**) and percentages (**D**) of GFP^+^ AML cells in the PB of mice at the indicated timepoints. Each symbol represents an individual mouse (*n* = 8). **E,** Schematic representation of drug treatment for the analysis of leukemia cell differentiation in the SP and BM. The number of GFP^+^ AML cells and percentages of c-kit^+^ blast cells in the SP (**F** and **G**) and BM (**H** and **I**). Each symbol represents the value in an individual mouse (*n* = 4). **, *P* < 0.01; *, *P* < 0.05; N.S., no significant difference using Tukey-Kramer test or log-rank test.

As MLL-AF9–positive AML cells are relatively sensitive to ATRA-mediated myeloid differentiation (36), we next explored the optimal drug conditions against human AML cell line carrying MLL-AF9. A 24-hour pre-exposure to ATRA+MRT was sufficient to induce irreversible cell growth arrest, irrespective of ATRA pretreatment (Supplementary Fig. S12a). Interestingly, cell proliferation after drug removal was suppressed even by short-term (6 hours) treatment with ATRA+MRT following ATRA pretreatment (Supplementary Fig. S12b). These *in vitro* data incited us to conduct a preclinical study to assess the therapeutic effects of combined treatment with ATRA and MRT in mice bearing MLL-AF9–positive AML. Drug administration commenced 6 days after the transplantation of primary AML mouse-derived cells, coinciding with the appearance of GFP^+^ (MLL-AF9^+^) cells in the PB of the recipients, as shown in the schedule (Fig. 5A). Although the combined treatment failed to cure AML and did not improve over the therapeutic effect of ATRA monotherapy on survival rate (Fig. 5B), it did lead to a significant reduction in total white blood cells (WBC) and GFP^+^ leukemia cells in the PB when compared with untreated or ATRA monotherapy groups (Fig. 5C and D). As the spleen (SP) and BM are major organs where leukemia cells propagate from blast cells, we examined the effects of combined treatment on cell composition in these organs (Fig. 5E). ATRA+MRT reduced total leukemia cells and the proportion of c-kit^+^ blast cells in the SP (Fig. 5F and G), but not in the BM (Fig. 5H and I). These negligible effects on BM may account for the insufficient therapeutic effect in mice bearing AML. In addition, MRT did not further depress the proportion of intrasplenic GFP^−^c-kit^+^ normal progenitor cells, which were slight reduced by ATRA. Similarly, GFP^−^Ly6G^+^ normal myeloid cells, minimally impacted by ATRA, are hardly affected by this treatment, indicating its tolerability in the normal hematopoietic system (Supplementary Fig. S13).

## Discussion

Myeloid differentiation is arrested by various oncogenic mutations in the early stages of malignant transformation, promoting the uncontrollable proliferation of blast cells in AML. Thus, differentiation therapy is a rational strategy to improve the prognosis of AML. However, leukemia cells undergoing differentiation often reacquire leukemogenic properties through dedifferentiation after the cessation of drug treatment (10). Here, we induced irreversible leukemia cell differentiation by pharmacologically inhibiting autophagosome formation in combination with conventional differentiation therapy. Mechanistically, blocking autophagic clearance resulted in the accumulation of cytosolic dsDNA fragments, activating the AIM2 inflammasome-caspase-1 pathway and consequently inhibiting p21 proteasomal degradation, which promoted myeloid differentiation and cell growth arrest in leukemia cells.

The G_1_ cell cycle stage is critical for determining whether hematopoietic cells will differentiate or proliferate. Thus, the role of the Cdk inhibitors, p21 and p27, in hematopoietic differentiation has been widely studied because of their regulatory function in the G_1_–S transition (37). Indeed, numerous studies have reported the crucial contribution of p21 to myeloid cell differentiation, including ATRA-induced APL cell differentiation (30, 38). Moreover, Goswami and colleagues (39) recently reported that the pharmacologic activation of protein phosphatase 2A enforces p21-dependent terminal differentiation in various types of myeloid leukemia cells. Furthermore, p21 can directly interact with myeloid differentiation-related transcription factors, such as C/EBPα (40) and STAT3 (41), to modulate their molecular function reciprocally. Thus, we propose that AIM2 inflammasome-mediated accumulation of p21 can turn differentiation therapy into an irreversible process in leukemia cells.

Furthermore, we observed that AIM2 inflammasome activation by combined treatment with ATRA and MRT crucially reduced the expression of USP10, which can stabilize SKP2 (32), an essential modulator of p21 under cell cycle progression (31). Agard and colleagues (33) comprehensively investigated the substrates for inflammatory caspases and identified many proteins that can be cleaved by caspase-1, including USP10. Thus, caspase-1 can modify the molecular function of numerous proteins, including well-known substrates such as IL1β and gasdermin D, through enzymatic cleavage upon inflammasome activation. Furthermore, Chadha and colleagues (42) recently reported that caspase-1 can directly degrade the ubiquitin ligase scaffold proteins, cullin-1 and cullin-5, thereby depressing the ubiquitylation activity of the RING E3 ligase complex. Likewise, caspase-1 may be able to directly degrade USP10 through enzymatic cleavage.

Here, combined treatment with ATRA and MRT induced a significant increase in the protein and mRNA expression of p21; however, the molecular mechanism whereby p21 is transcriptionally regulated remains elusive. The transcription of p21 appears to be controlled by both p53-dependent and -independent mechanisms (43). A certain p53-independent pathway may enhance p21 transcription in the HL-60 cell line, which is a p53-null cell line. Accumulating studies have unraveled the contribution of several transcription factors, such as Sp1, Sp3, AP2, STATs, C/EBPα, and C/EBPβ, to the p53-independent p21 transcriptional regulation (43); however, most of them are also involved in the differentiation of hematopoietic cells. Thus, the initial molecular event triggering p21 transcription during myeloid differentiation remains unclear.

The most frequently mutated gene in AML is *FLT3*, which is classified into two distinct alterations, either FLT3-ITD or point mutations in the tyrosine kinase domain. Radomska and colleagues (44) recently reported that FLT3 activation by its genetic mutation inhibits C/EBPα-induced myeloid differentiation through ERK1/2-mediated phosphorylation, and that FLT3 inhibitors can promote differentiation in leukemia cells with *FLT3* mutations. Here, we demonstrate that MRT treatment can synergize with the FLT3 inhibitor, quizartinib, to induce the irreversible differentiation of FLT3-ITD^+^ AML cell line, similarly to what was observed in ATRA-mediated differentiation. Collectively, the evidence suggests that the inhibition of autophagosome formation may potentially complement the clinical effects of molecular targeted therapy against FLT3 mutation–induced AML by promoting leukemia cell differentiation.

Achieving sufficient therapeutic doses of anticancer agents inside the BM through systemic administration is arguably difficult (45). Indeed, we observed that *in vivo* administration of ATRA and MRT could reduce the total counts of leukemia cells and the proportion of c-kit^+^ blast cells in the SP. In contrast, the treatment was less effective in the BM, suggesting that the drug may have failed to reach a sufficient concentration in the BM. Many studies have developed novel strategies for drug delivery to the BM (45). Thus, we believe that further improvements in the administration of these drugs and the engineering of pharmacologic devices with optimized drug delivery to the BM will pave the way for completing AML differentiation therapy.

## Supplementary Material

Supplementary Figure 1 Fig. S1 and its legend

Supplementary Figure 2 Fig. S2 and its legend

Supplementary Figure 3 Fig. S3 and its legend

Supplementary Figure 4 Fig. S4 and its legend

Supplementary Figure 5 Fig. S5 and its legend

Supplementary Figure 6 Fig. S6 and its legend

Supplementary Figure 7 Fig. S7 and its legend

Supplementary Figure 8 Fig. S8 and its legend

Supplementary Figure 9 Fig. S9 and its legend

Supplementary Figure 10 Fig. S10 and its legend

Supplementary Figure 11 Fig. S11 and its legend

Supplementary Figure 12 Fig. S12 and its legend

Supplementary Figure 13 Fig. S13 and its legend
