# Supplementary material for: Autophagy Inhibition–induced Cytosolic DNA Sensing Combined with Differentiation Therapy Induces Irreversible Myeloid Differentiation in Leukemia Cells
Source: Cancer Res Commun. 2024 Mar 20;4(3):849–60. doi: 10.1158/2767-9764.CRC-23-0507 (PMC10953625; doi:10.1158/2767-9764.CRC-23-0507)
Supplement: Supplementary Figure 1 — Fig. S1 and its legend [file crc-23-0507-s01.pdf]

Supplementary Figure 1. Apoptosis induction in HL-60 cells. Apoptosis analysis of HL-60 cells 24 h after treatment with 1 μM ATRA, 1 μM MRT, or ATRA+MRT. Representative results of FITC-conjugated annexin V and PI staining from three independent experiments are shown here. Percentages of PI^+ annexin V^-, PI^+ annexin V^+, and PI^- annexin V^+ cells among R1-gated cells were determined.

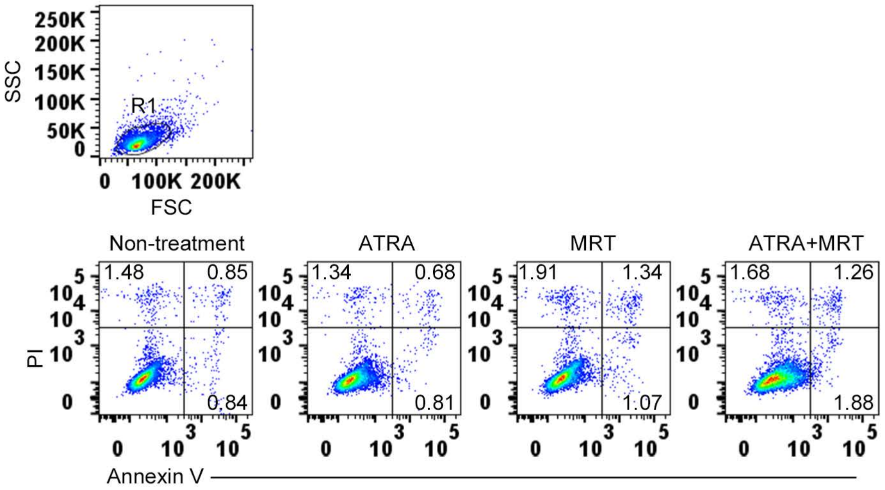

Supplementary Figure 1
