# Supplementary material for: Autophagy Inhibition–induced Cytosolic DNA Sensing Combined with Differentiation Therapy Induces Irreversible Myeloid Differentiation in Leukemia Cells
Source: Cancer Res Commun. 2024 Mar 20;4(3):849–60. doi: 10.1158/2767-9764.CRC-23-0507 (PMC10953625; doi:10.1158/2767-9764.CRC-23-0507)
Supplement: Supplementary Figure 2 — Fig. S2 and its legend [file crc-23-0507-s02.pdf]

Supplementary Figure 2. Combined treatment with SBI and ATRA in HL-60 cells. (a) NBT staining of HL-60 cells 24 h after treatment with 1 μM ATRA, 1 μM MRT, 10 μM SBI, ATRA+MRT, or ATRA+SBI. Non-treated cells were used as a control. Representative results from three independent experiments are shown here**. (b)** Cell proliferation of HL-60 cells in drug-free medium after ATRA, SBI, or ATRA+SBI treatment for 24 h (n = 4). Fold change in cell number was calculated by dividing the values at each time point with the values at 0 d.

a

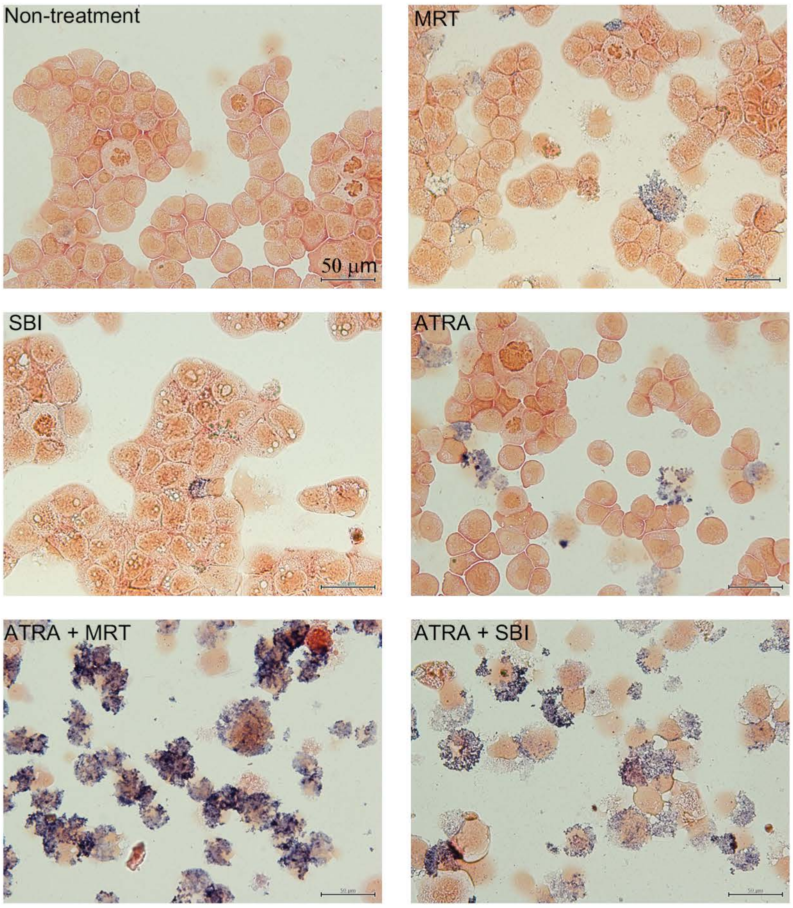

b

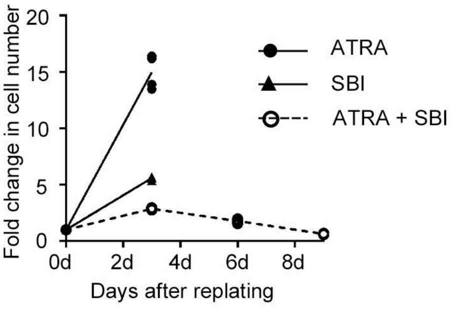

Supplementary Figure 2
