# Supplementary material for: Autophagy Inhibition–induced Cytosolic DNA Sensing Combined with Differentiation Therapy Induces Irreversible Myeloid Differentiation in Leukemia Cells
Source: Cancer Res Commun. 2024 Mar 20;4(3):849–60. doi: 10.1158/2767-9764.CRC-23-0507 (PMC10953625; doi:10.1158/2767-9764.CRC-23-0507)
Supplement: Supplementary Figure 3 — Fig. S3 and its legend [file crc-23-0507-s03.pdf]

Supplementary Figure 3. Induction of irreversible differentiation by combined treatment with ATRA and MRT in human myeloid leukemia cell lines. Cell proliferation of THP-1, K562, and KG-1 cells in drug-free medium after treatment with 1 μM ATRA, 1 μM MRT, or ATRA+MRT for 48 h (*n* = 4). Untreated cells were used as a control. Fold change in cell number was calculated by dividing the values at each time point with the values at 0 d.

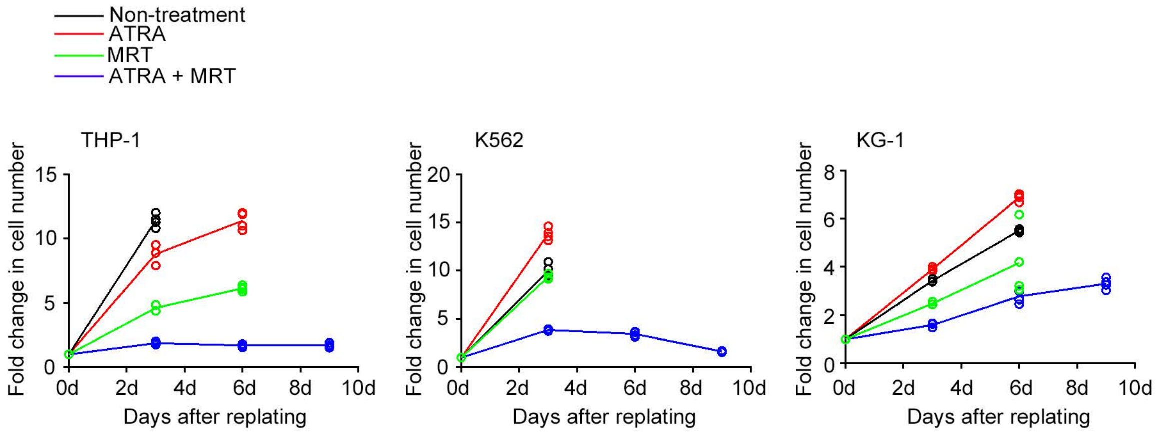

Supplementary Figure 3
