# Supplementary material for: Autophagy Inhibition–induced Cytosolic DNA Sensing Combined with Differentiation Therapy Induces Irreversible Myeloid Differentiation in Leukemia Cells
Source: Cancer Res Commun. 2024 Mar 20;4(3):849–60. doi: 10.1158/2767-9764.CRC-23-0507 (PMC10953625; doi:10.1158/2767-9764.CRC-23-0507)
Supplement: Supplementary Figure 4 — Fig. S4 and its legend [file crc-23-0507-s04.pdf]

Supplementary Figure 4. Alteration in the expression of leukocyte activation- and cell cycle-related genes by combined treatment with ATRA and MRT. Relative mRNA expression of the indicated genes in HL-60 cells 48 h after ATRA, MRT, or ATRA+MRT treatment. Untreated cells were used as a control. Data represent the mean ± SD from three independent experiments. $^{**}P < 0.01$ using Tukey-Kramer test.

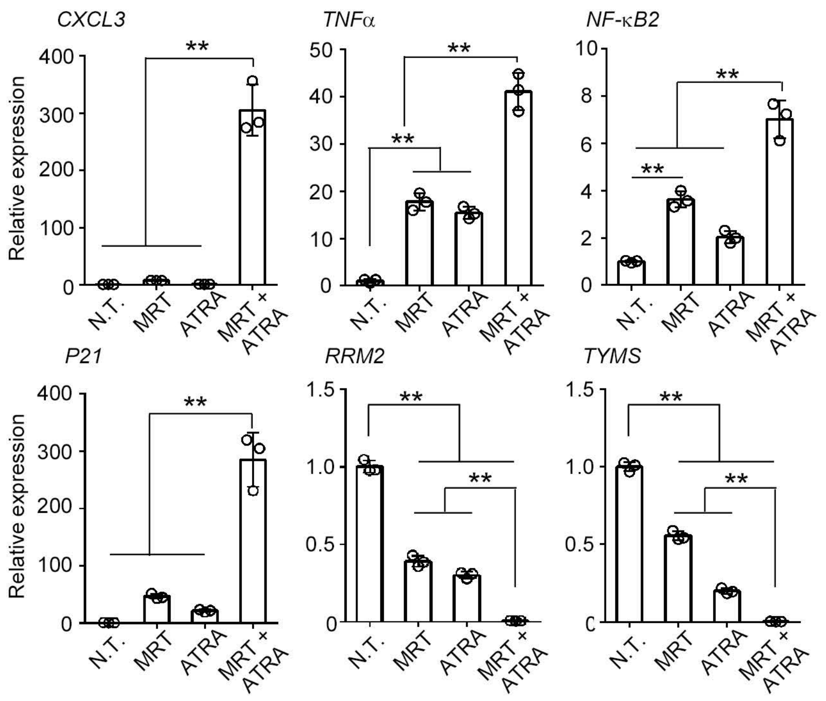

Supplementary Figure 4
