# Supplementary material for: Autophagy Inhibition–induced Cytosolic DNA Sensing Combined with Differentiation Therapy Induces Irreversible Myeloid Differentiation in Leukemia Cells
Source: Cancer Res Commun. 2024 Mar 20;4(3):849–60. doi: 10.1158/2767-9764.CRC-23-0507 (PMC10953625; doi:10.1158/2767-9764.CRC-23-0507)
Supplement: Supplementary Figure 5 — Fig. S5 and its legend [file crc-23-0507-s05.pdf]

Supplementary Figure 5. AIM2 KD modulates the ATRA+MRT combined treatment-mediated changes in the mRNA expression of leukocyte activation- and cell cycle-related genes. Relative mRNA expression of the indicated genes in shControl- and shAIM2-transduced HL-60 cells 24 h after ATRA+MRT or ATRA treatment. Data represent the mean ± SD from three independent experiments. $^{**}P < 0.01$ using two-sided Student's *t*-test.

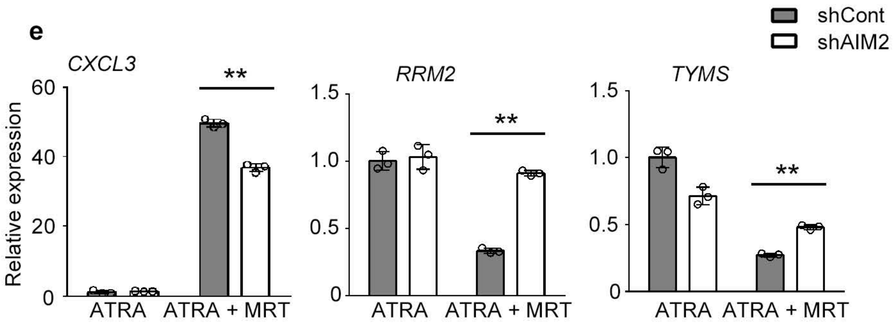

Supplementary Figure 5
