# Supplementary material for: Autophagy Inhibition–induced Cytosolic DNA Sensing Combined with Differentiation Therapy Induces Irreversible Myeloid Differentiation in Leukemia Cells
Source: Cancer Res Commun. 2024 Mar 20;4(3):849–60. doi: 10.1158/2767-9764.CRC-23-0507 (PMC10953625; doi:10.1158/2767-9764.CRC-23-0507)
Supplement: Supplementary Figure 6 — Fig. S6 and its legend [file crc-23-0507-s06.pdf]

Supplementary Figure 6. Blunted upregulation of p21 protein but not its mRNA by AIM KD. Expression of mRNA **(a)** and protein **(b)** of p21 in shControl- and shAIM2-1 transduced HL-60 cells 24 h after ATRA+MRT or ATRA treatment. Representative results and MFI are shown (b).

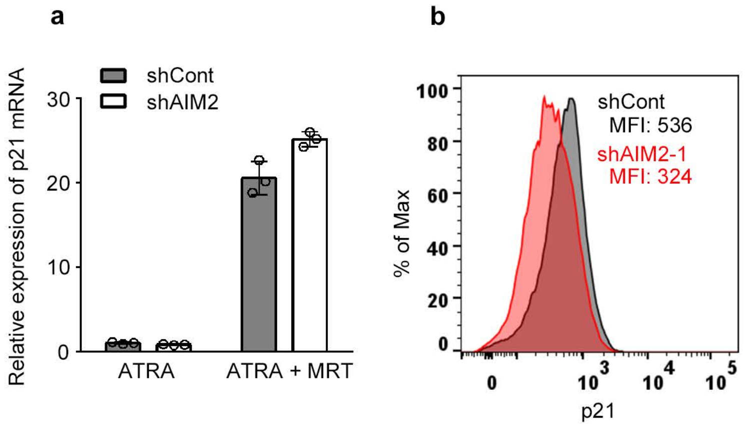

Supplementary Figure 6
