# Supplementary material for: Autophagy Inhibition–induced Cytosolic DNA Sensing Combined with Differentiation Therapy Induces Irreversible Myeloid Differentiation in Leukemia Cells
Source: Cancer Res Commun. 2024 Mar 20;4(3):849–60. doi: 10.1158/2767-9764.CRC-23-0507 (PMC10953625; doi:10.1158/2767-9764.CRC-23-0507)
Supplement: Supplementary Figure 7 — Fig. S7 and its legend [file crc-23-0507-s07.pdf]

Supplementary Figure 7. Inhibition of myeloid differentiation by AIM KD in THP-1 cells. Expression of CD11b **(a)** and p21 **(b)** in shControl- and shAIM2-transduced THP-1 cells after combined treatment with 1 μM ATRA and 1 μM MRT for 48 h. Fold increases in CD11b expression were calculated by dividing the values of treated cells with those of non-treated cells. Data represent the mean ± SD from three independent experiments. $^{**}P < 0.01$ using Tukey-Kramer test (a). Representative results and mean ± SD of MFI from three independent experiments are shown here. $^{**}P < 0.01$ using Dunnett test (b).

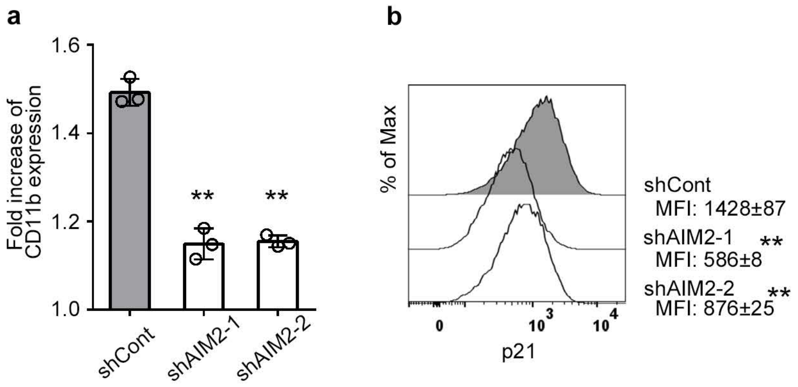

Supplementary Figure 7
