# Supplementary material for: Autophagy Inhibition–induced Cytosolic DNA Sensing Combined with Differentiation Therapy Induces Irreversible Myeloid Differentiation in Leukemia Cells
Source: Cancer Res Commun. 2024 Mar 20;4(3):849–60. doi: 10.1158/2767-9764.CRC-23-0507 (PMC10953625; doi:10.1158/2767-9764.CRC-23-0507)
Supplement: Supplementary Figure 8 — Fig. S8 and its legend [file crc-23-0507-s08.pdf]

Supplementary Figure 8. Validation of p21 KD in HL-60 cells. p21 expression in shControl- and shp21-transduced HL-60 cells, which were incubated in the presence or absence of 1 μM ATRA and 1 μM MRT for 48 h. MFI of a single experiment is shown here.

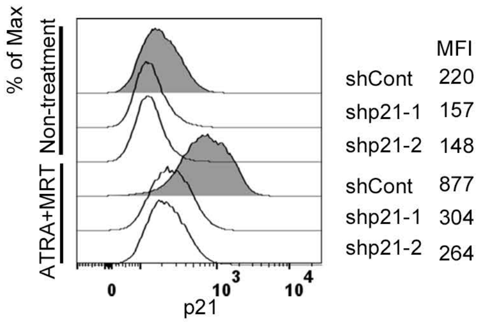

Supplementary Figure 8
