# Supplementary material for: Autophagy Inhibition–induced Cytosolic DNA Sensing Combined with Differentiation Therapy Induces Irreversible Myeloid Differentiation in Leukemia Cells
Source: Cancer Res Commun. 2024 Mar 20;4(3):849–60. doi: 10.1158/2767-9764.CRC-23-0507 (PMC10953625; doi:10.1158/2767-9764.CRC-23-0507)
Supplement: Supplementary Figure 9 — Fig. S9 and its legend [file crc-23-0507-s09.pdf]

Supplementary Figure 9. Morphological changes in MOLM-14 cells after combined treatment with quizartinib and MRT. Giemsa and NBT staining of MOLM-14 cells 24 and 48 h after treatment with 5 nM quizartinib or 5 nM quizartinib and 1 μM MRT in the presence of 10 ng/ml FGF2. Representative results from three independent experiments are shown here.

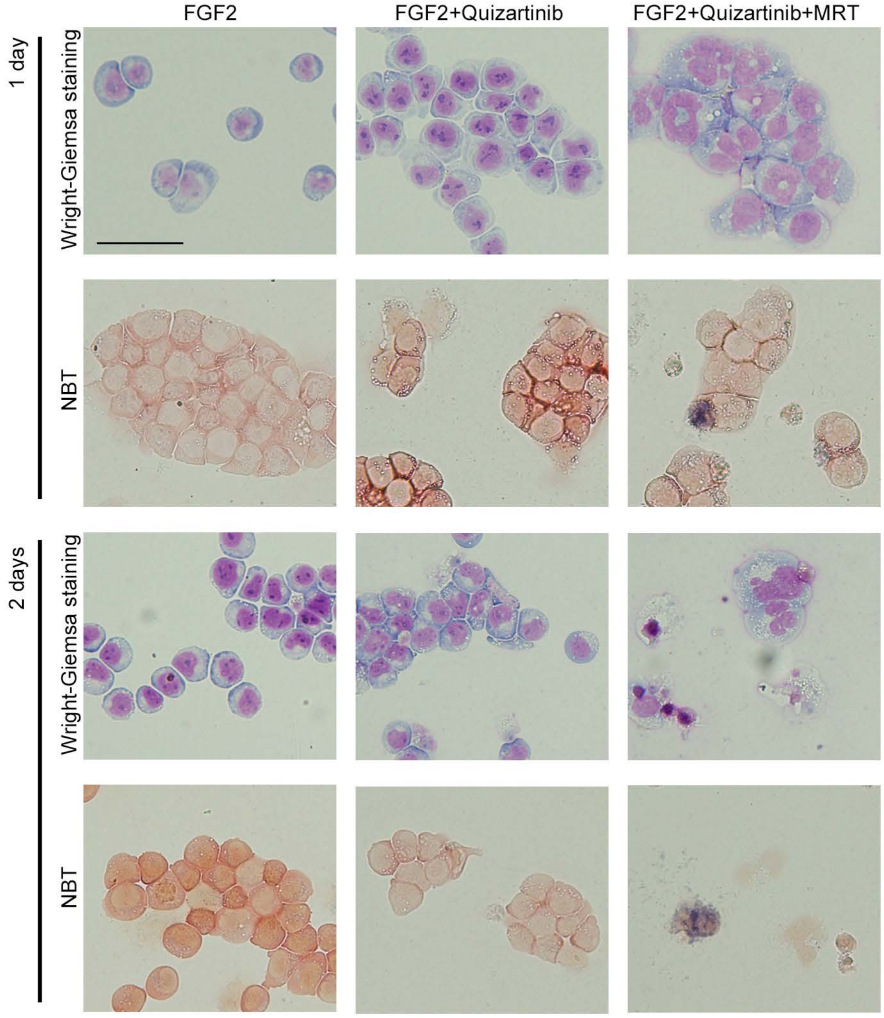

Supplementary Figure 9
