# Supplementary material for: Autophagy Inhibition–induced Cytosolic DNA Sensing Combined with Differentiation Therapy Induces Irreversible Myeloid Differentiation in Leukemia Cells
Source: Cancer Res Commun. 2024 Mar 20;4(3):849–60. doi: 10.1158/2767-9764.CRC-23-0507 (PMC10953625; doi:10.1158/2767-9764.CRC-23-0507)
Supplement: Supplementary Figure 10 — Fig. S10 and its legend [file crc-23-0507-s10.pdf]

Supplementary Figure 10. AIM KD in MOLM-14 cells. (a) AIM2 expression in shControl- and shAIM2-transduced MOLM-14 cells. MFI of a single experiment is shown here. Expression of p21 **(b)** and Giemsa staining **(c)** of shControl- and shAIM2-transduced MOLM-14 cells after combined treatment with 5 nM quizartinib and 1 μM MRT in the presence of 10 ng/ml FGF2. Representative results and mean ± SD of MFI from three independent experiments are shown here**.** *$P < 0.05$ using two-sided Student's *t*-test.

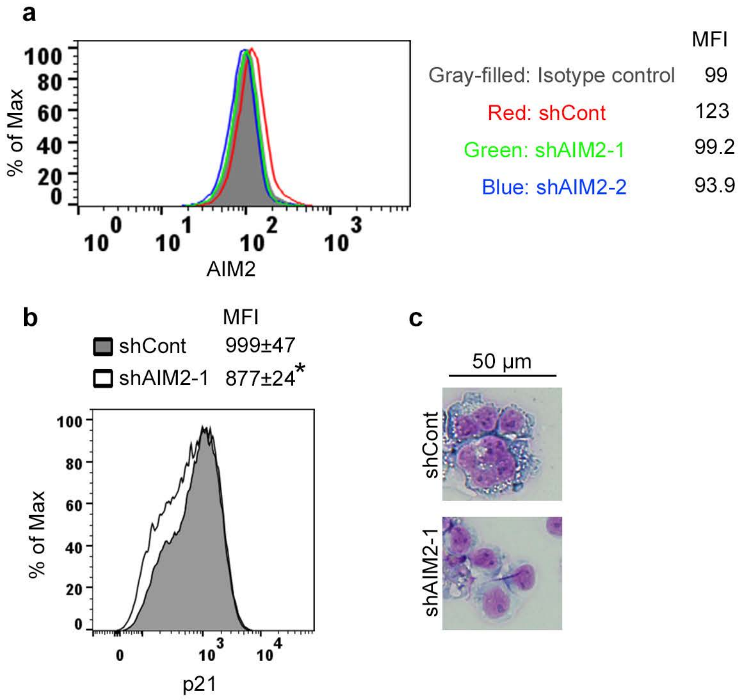

Supplementary Figure 10
