# Supplementary material for: Autophagy Inhibition–induced Cytosolic DNA Sensing Combined with Differentiation Therapy Induces Irreversible Myeloid Differentiation in Leukemia Cells
Source: Cancer Res Commun. 2024 Mar 20;4(3):849–60. doi: 10.1158/2767-9764.CRC-23-0507 (PMC10953625; doi:10.1158/2767-9764.CRC-23-0507)
Supplement: Supplementary Figure 11 — Fig. S11 and its legend [file crc-23-0507-s11.pdf]

Supplementary Figure 11. Cell cycle arrest and myeloid differentiation in MOLM-14 cells in the absence of FGF2. MOLM-14 cells were treated with quizartinib or quizartinib+MRT for 24 h. **(a)** Cell growth after drug-removal (n = 4), **(b)** Giemsa staining (n = 3), and **(c)** CD38 expression (n = 3) were determined. Representative results and mean ± SD of MFI from three independent experiments are shown here**.** **$P < 0.01$ using Dunnett test.

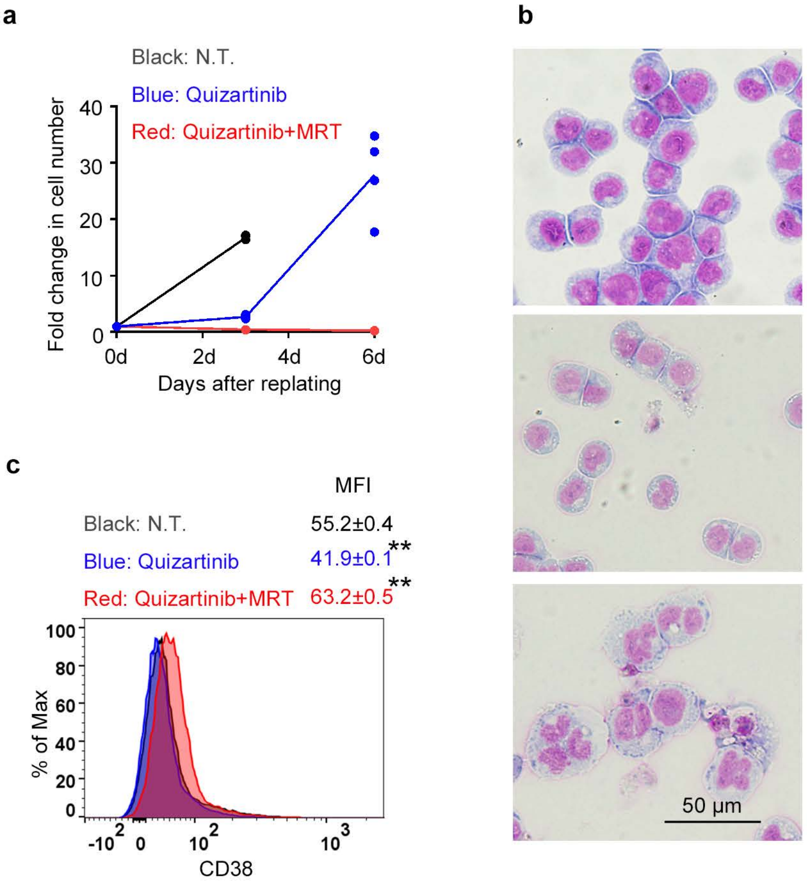

Supplementary Figure 11
