# Supplementary material for: Autophagy Inhibition–induced Cytosolic DNA Sensing Combined with Differentiation Therapy Induces Irreversible Myeloid Differentiation in Leukemia Cells
Source: Cancer Res Commun. 2024 Mar 20;4(3):849–60. doi: 10.1158/2767-9764.CRC-23-0507 (PMC10953625; doi:10.1158/2767-9764.CRC-23-0507)
Supplement: Supplementary Figure 12 — Fig. S12 and its legend [file crc-23-0507-s12.pdf]

Supplementary Figure 12. ATRA-pretreatment potentiates the effects of combined treatment with ATRA and MRT. Cell proliferation of MOLM-13 cells in drug-free medium was determined 24 h **(a)** and 6 h **(b)** after combined incubation with 1 μM ATRA and 1 μM MRT following exposure to 1 μM ATRA for 48 h (*n* = 4). Fold change in cell number was calculated by dividing the values at each time point with the values at 0 d.

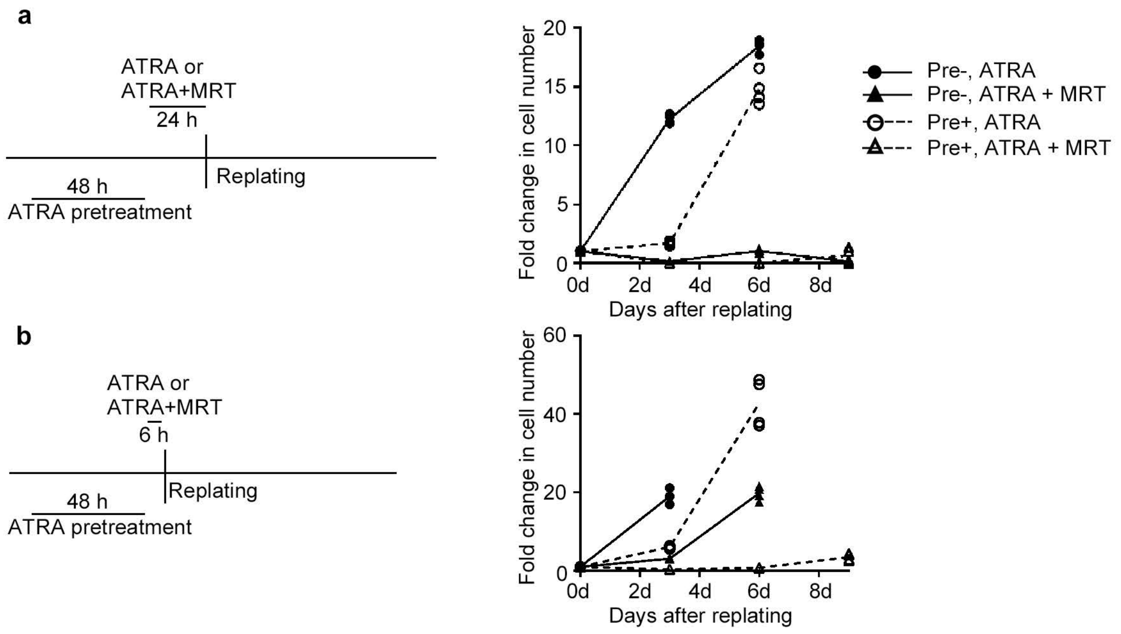

Supplementary Figure 12
