# Supplementary material for: Autophagy Inhibition–induced Cytosolic DNA Sensing Combined with Differentiation Therapy Induces Irreversible Myeloid Differentiation in Leukemia Cells
Source: Cancer Res Commun. 2024 Mar 20;4(3):849–60. doi: 10.1158/2767-9764.CRC-23-0507 (PMC10953625; doi:10.1158/2767-9764.CRC-23-0507)
Supplement: Supplementary Figure 13 — Fig. S13 and its legend [file crc-23-0507-s13.pdf]

Supplementary Figure 13. Marginal effect of combined treatment with ATRA and MRT in the normal hematopoietic system. Mice bearing MLL-AF9-positive AML were treated with MRT in combination with ATRA, as shown in the schedule (Figure 5e). Percentages of $GFP^{-}Ly6G^{+}$ normal myeloid cells **(a)** and GFP^{-}c-kit^{+} normal progenitor cells **(b)** in the SP are shown here. Each symbol represents the value in an individual mouse (n = 4). *P < 0.05; N.S., no significant difference using Tukey-Kramer test.

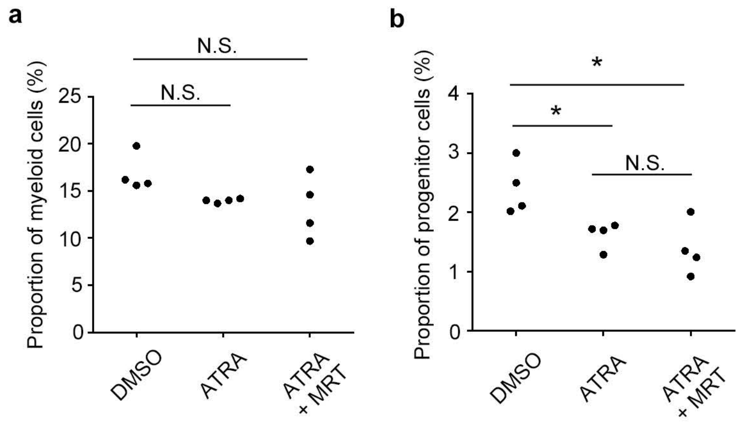

Supplementary Figure 13
